# RNA Interference of Endochitinases in the Sugarcane Endophyte *Trichoderma virens* 223 Reduces Its Fitness as a Biocontrol Agent of Pineapple Disease

**DOI:** 10.1371/journal.pone.0047888

**Published:** 2012-10-23

**Authors:** Aline S. Romão-Dumaresq, Welington Luiz de Araújo, Nicholas J. Talbot, Christopher R. Thornton

**Affiliations:** 1 Department of Genetics, Escola Superior de Agricultura “Luiz de Queiroz”, University of São Paulo, Piracicaba, São Paulo, Brazil; 2 Department of Microbiology, Institute of Biological Sciences, University of São Paulo, São Paulo, São Paulo, Brazil; 3 Biosciences, College of Life and Environmental Sciences, University of Exeter, Exeter, United Kingdom; University of Wisconsin-Milwaukee, United States of America

## Abstract

The sugarcane root endophyte *Trichoderma virens* 223 holds enormous potential as a sustainable alternative to chemical pesticides in the control of sugarcane diseases. Its efficacy as a biocontrol agent is thought to be associated with its production of chitinase enzymes, including *N*-acetyl-ß-D-glucosaminidases, chitobiosidases and endochitinases. We used targeted gene deletion and RNA-dependent gene silencing strategies to disrupt *N*-acetyl-ß-D-glucosaminidase and endochitinase activities of the fungus, and to determine their roles in the biocontrol of soil-borne plant pathogens. The loss of *N*-acetyl-ß-D-glucosaminidase activities was dispensable for biocontrol of the plurivorous damping-off pathogens *Rhizoctonia solani* and *Sclerotinia sclerotiorum,* and of the sugarcane pathogen *Ceratocystis paradoxa,* the causal agent of pineapple disease. Similarly, suppression of endochitinase activities had no effect on *R. solani* and *S. sclerotiorum* disease control, but had a pronounced effect on the ability of *T. virens* 223 to control pineapple disease. Our work demonstrates a critical requirement for *T. virens* 223 endochitinase activity in the biocontrol of *C. paradoxa* sugarcane disease, but not for general antagonism of other soil pathogens. This may reflect its lifestyle as a sugarcane root endophyte.

## Introduction

Sugarcane is an economically important crop that is grown in more than 100 tropical countries, contributing not only to economic growth and development but also to global energy security in the form of sugarcane-fermented bioethanol. In 2010, the sugarcane sector contributed US$50 billion to the gross domestic product of Brazil, equivalent to almost 2.4% of the entire economy [1]. ‘Pineapple’ disease of sugarcane is caused by the soil-borne fungus *Ceratocystis paradoxa* (Dade) and is a devastating disease that causes complete loss of sugarcane setts, and occurs in almost all countries where sugarcane is grown. The disease affects sugarcane in the first week of planting and can reduce the germination of setts by up to 47% and subsequent cane yields by 31–35% [2]. Control of the disease is considered a priority especially where soil inoculum levels are high. Indeed, procedures that favour germination of buds and emergence of young shoots can dramatically improve crop yields. At present, systemic fungicides are used to protect against *C. paradoxa*, but chemical control is expensive and environmentally damaging. Sustainable methods of disease control are therefore urgently needed.

Biological control using beneficial soil microorganisms represents a sustainable means by which soil-borne pathogens can be controlled and *Trichoderma* species have been proposed as credible alternatives to environmentally-damaging chemicals in the control of plant diseases [3], [4]. Soil applications of *Trichoderma harzianum*, for example, have been found to antagonise *C. paradoxa* leading to increased germination of sugarcane setts and improvements in cane yields [2]. In previous work, we have demonstrated that the sugarcane root endophyte *T. virens* 223 is an effective antagonist of the pathogen [5], but the mechanisms of biocontrol of sugarcane pineapple disease by the fungus are currently poorly understood. *Trichoderma virens* is considered one of the most effective biological control agents studied to date [6], and its ability to control plant diseases has been attributed to its production of anti-fungal compounds [7]–[11] and induction of plant disease resistance [12], [13]. The contribution of chitinolytic enzymes to the anti-fungal activity of *T. virens* is less well understood, but chitinolytic enzymes are now regarded as key components of biocontrol by *Trichoderma* species.

Chitin is an integral component of the hyphal cell walls of fungal plant pathogens [14], and its enzymatic degradation allows subsequent colonization of host tissues by *Trichoderma* species [3], [15]. Chitinases are separated into different groups depending on their modes of action [16]. While *N*-acetyl-ß-D-glucosaminidases (EC 3.2.1.52) belong to glycoside hydrolase (GH) family 20 and catalyze the release of terminal, non-reducing N-acetylglucosamine (GlcNAc) residues from chitin, fungal endochitinases (EC 3.2.1.14) are members of GH family 18 and catalyze hydrolysis of the ß-1,4 linkages in chitin and chito-oligomers [16]. Although several *Trichoderma* chitinases have been purified and characterized [9], [17]–[24], and some of the corresponding genes cloned [24]–[32], there have been conflicting reports regarding the roles of these enzymes in biological control activities of *Trichoderma* species. Many studies have, for instance, ascribed biocontrol efficacies to chitinase production [9], [30], [33]–[38], while others have shown that biocontrol activities are governed by mechanisms other than chitinase production [39]–[42].

Recently, genome analysis of the *T. virens* strain Gv29-8 has shown that the fungus possesses two *N*-acetyl-ß-D-glucosaminidase-encoding genes and one additional hypothetical protein, distantly related to *N*-acetyl-ß-hexosaminidase [43]. In contrast, *T. virens* Gv29-8 contains 36 chitinase-encoding genes, the highest number yet reported in a fungal genome [43]. This high degree of redundancy therefore makes investigations of the involvement of chitinase in *T. virens* biocontrol very challenging. To date, only one endochitinase gene (*T. harzianum* cht42 = *T. harzianum* ech42 = *T. virens* ech1) has been functionally characterized. The endochitinase gene cht42 of *T. harzianum* was over-expressed and disrupted, resulting in enhanced and reduced biocontrol, respectively, of the root pathogen *Rhizoctonia solani* [44]. Transgenic cotton plants expressing the *T. virens* cht42 endochitinase gene were also shown to be more resistant to disease [15], [45], [46]. Despite these studies, the role of chitinolytic enzymes in the biocontrol capability of *T. virens* remains unclear.

In this study, we set out to investigate the role of *N*-acetyl-ß-D-glucosaminidases and endochitinase enzymes in the biological control of ‘pineapple’ disease of sugarcane by *T. virens* 223. We used targeted gene deletion analysis to study the function of two *N*-acetyl-ß-D-glucosaminidase-encoding genes and RNA-dependent gene silencing to impair the activity of the expanded subgroup A chitinase gene family of *T. virens*. We report that the biocontrol efficacy of *T. virens* is unaffected by loss of *N*-acetyl-ß-D-glucosaminidase activity, but that impairing endochitinase activity by gene silencing resulted in dramatically reduced fitness of *T. virens* as a biocontrol agent of sugarcane pineapple disease. Significantly, the ability of *T. virens* to control diseases caused by other soil-borne fungi was unaffected. When considered together, this indicates that endochitinases play a critical function in *T. virens* as a biocontrol agent of sugarcane disease but not as a general antagonist of other soil pathogens. This may reflect its lifestyle as a sugarcane-specific root endophyte.

## Materials and Methods

### Fungal Strains and Culture Conditions

The sugarcane root endophyte *Trichoderma virens* strain *Tv*.223 (GenBank accession number GQ495269) has been previously described [5], [47], [48]. *Ceratocystis paradoxa*, the causal agent of sugarcane pineapple disease, was supplied by Centro de Tecnologia Canavieira. The pathogen *Sclerotinia sclerotiorum* (GenBank accession number FJ984493) was obtained from the University of Exeter culture collection [49] and the *Rhizoctonia solani* strain was obtained from the Genetics of Microorganisms Laboratory (Department of Genetics/ESALQ, University of São Paulo) culture collection. Fungi were grown on potato dextrose agar (PDA, Difco) at 26°C under a 16 h photoperiod of fluorescent light.

### DNA Extraction, Amplification and Sequencing of *Tv.*223 Chitinase Genes

Genomic DNA of *T. virens* strain 223 was prepared according to the method of [50]. PCR primers were designed for amplification of the two *N*-acetyl-ß-D-glucosaminidase-encoding genes *Tvnag1* and *Tvnag2* and the three endochitinase-encoding genes *Tvech1, Tvech2* and *Tvech3* (Table 1) using the *T. virens* Gv29-8 v2.0 genome available at the Joint Genome Institute (JGI) Genome Portal (http://genome.jgi-psf.org/TriviGv29_8_2/TriviGv29_8_2.home.html) as reference. PCR products were purified with the UltraClean PCR Clean-up Kit (MoBio Laboratories Inc.) and sequenced at the Human Genome Research Center, University of São Paulo, Brazil, using an automated ABI 3730 DNA Analyser (Applied Biosystems). The gene fragments were assembled using the CAP3 Sequence Assembly Program [51] and the chitinolytic function was confirmed by a BLAST search for similar sequences and also an InterPro [52] search for chitinolytic enzyme motif signatures. *Trichoderma virens* strain 223 genes sequenced in this work were deposited at GenBank under accession numbers JQ066769 to JQ066773.

**Table 1:** Details of PCR primers used in this study.

Primer name	Sequence 5′-3′	Target
Tvech1-Fw	Cagcacagaagtggcaagcttgaa	*Tvech1* gene
Tvech1-Rv	acttggtacacacacgaattcacc	*Tvech1* gene
Tvech1-Rv2	tccgttgtaagtctggccaatacc	*Tvech1* gene
Tvech2-Fw	ccatctcaagtaaacatgtcggga	*Tvech2* gene
Tvech2-Rv	gggtgcaaacaacaagtaagcctc	*Tvech2* gene
Tvech2-Rv2	ttgtgggttatagcagtagctgga	*Tvech2* gene
Tvech3-Fw	gaccagaacctactttgaaggctt	*Tvech3* gene
Tvech3-Rv	gcctcgcagaaccatacaagacaa	*Tvech3* gene
Tvech3-Rv2	ttgcccagatacagtatcccagct	*Tvech3* gene
Tvnag1-Fw	cgtagtagtgctattgccatcgct	*Tvnag1* gene
Tvnag1-Fw2	gatactacccagacgttcaagccg	*Tvnag1* gene
Tvnag1-Rv	tcccggcttctcttaatccatacc	*Tvnag1* gene
Tvnag1-Rv2	acggccgcagtccaagtactataa	*Tvnag1* gene
Tvnag2-Fw	Tcgtgtggcctgaccttgtcaact	*Tvnag2* gene
Tvnag2-Fw2	tcttcttcaagcatagctcaggca	*Tvnag2* gene
Tvnag2-Rv	taccacactgcatactttgttccg	*Tvnag2* gene
Tvnag2-Rv2	ataaccttgaatccagcctccgcg	*Tvnag2* gene
HEX1_Fw	ccggttgctgccgaaaccttcaat	*Tvnag2* LF
HEX1.M13F_Rev	gtcgtgactgggaaaaccctggcggttgccggttgaggagtgtttcgg	*Tvnag2* LF
HEX2.M13R_Fw	tcctgtgtgaaattgttatccgctccgattacgcagctgtggtgtagt	*Tvnag2* RF
HEX2_Rev	ggccgaaatgatgaccctttgcgg	*Tvnag2* RF
Chito2_50.1	gcttcgactgctactgtactccgt	*Tvnag1* LF
Chito2_m13F	gtcgtgactgggaaaaccctggcggctgaaagccaatgcggcaatcgc	*Tvnag1* LF
Chito2_m13R	tcctgtgtgaaattgttatccgctcgctgttcacctaaggggagagtc	*Tvnag1* RF
Chito2_30.1	aacagcatacactgggaagactcg	*Tvnag1* RF
HY split	ggatgcctccgctcgaagta	*hph* gene
YG split	cgttgcaagacctgcctgaa	*hph* gene
IL split	tctggttgtattctcaggac	*ilv* gene
LV split	cataccaagcatgtgcagtg	*ilv* gene
M13 F	cgccagggttttcccagtcacgac	binding site of pUC/M13 forward sequencing primer
M13 R	agcggataacaatttcacacagga	binding site of pUC/M13 reverse sequencing primer
1F.endochi.XhoI	cgccaaatctagtctcgagct	*Tvech2* gene
2R.endochi.HindIII	aagcttacccctccccgatgccattat	*Tvech2* gene
3F.endochi.KpnI	ggtacccgccaaatctagtctcgagct	*Tvech2* gene
4R.endochi.SphI	gcatgcacccctccccgatgccattat	*Tvech2* gene
1F.SpeI.ToxA	actagtcatggaggagttctgtacgcgc	*ToxA* promoter
2R.fusion.ToxA	agcagctcgagactagatttggcggacctatattcattcaatgtcagc	*ToxA* promoter
3F.fusion.ToxA	gctgacattgaatgaatataggtccgccaaatctagtctcgagctgct	*Tvech2* gene
Hex probe F	accagacggtccaagttacctaca	*Tvnag2* gene
Hex probe R	tgtttgccttatactcgtcgcctc	*Tvnag2* gene
Chito2 probe F	cgtgctcttcattgaccaggctgt	*Tvnag1* gene
Chito2 probe R	gctcggagtcgttgacgttgagct	*Tvnag1* gene
Hyg probe 2F	aagcctgaactcaccgcgacgtct	*hph* gene
Hyg probe 3R	tgctggggcgtcggtttccactat	*hph* gene
Endochi probe1F	tgagcttcctcggcaaatccgtgg	*Tvech1* gene
Endochi probe1R	agcagaatccgtgttgaagggagt	*Tvech1* gene
Endochi probe2F	gggagacggctatcgttcagttgc	*Tvech2* gene
Endochi probe2R	cgaagatgccattctcccacgacc	*Tvech2* gene
Endochi probe3F	gctcctttgcgtcgttttggccgt	*Tvech3* gene
Endochi probe3R	gtgtcgccacggtcagaaggtagt	*Tvech3* gene

LF – left flank.

RF – right flank.

### Targeted Deletion of Chitinase Genes and Construction of pSilent-1 Silencing Vectors

**Figure 1:**
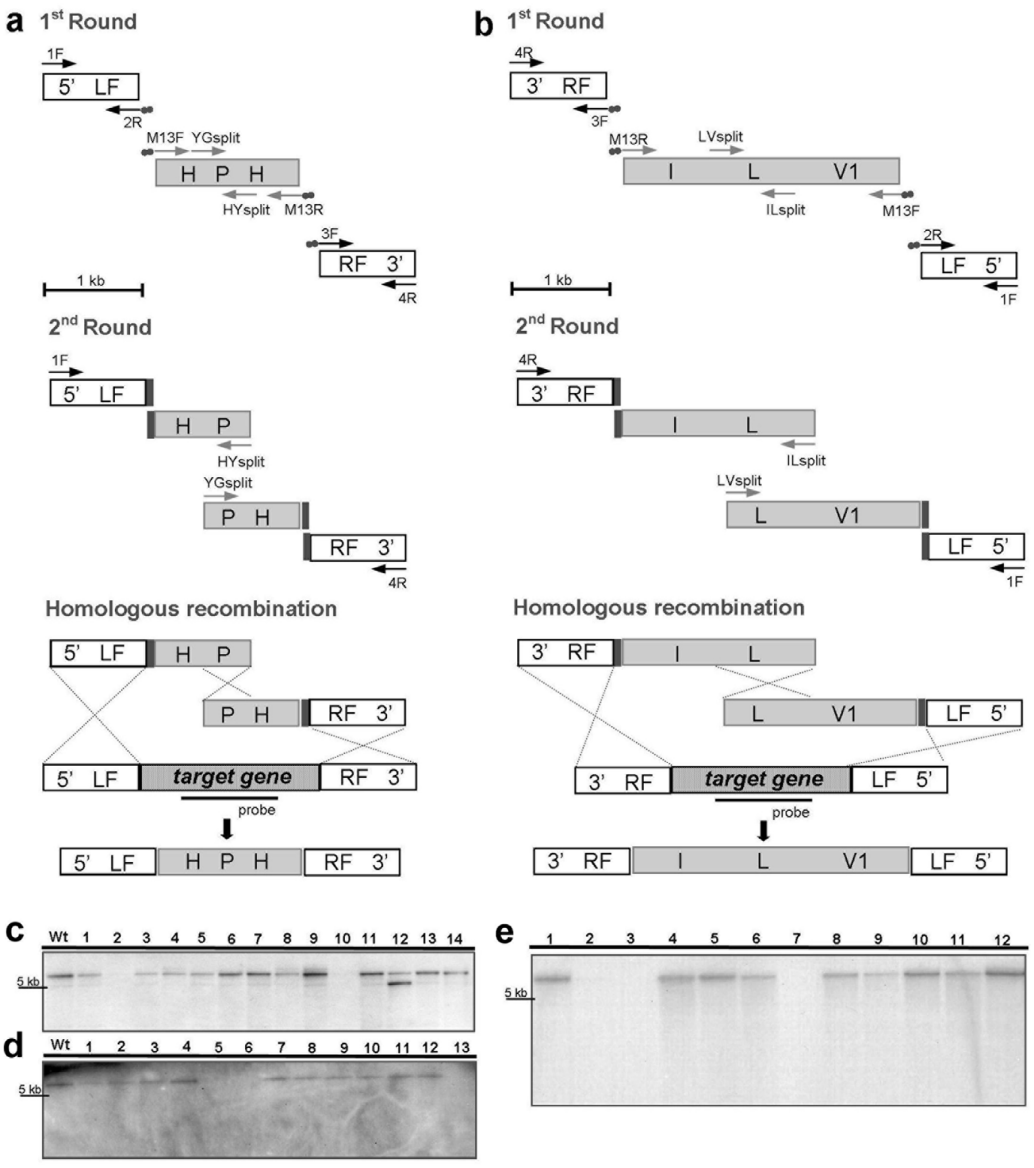
Targeted gene deletion of the *N-*acetyl-ß-D-glucosaminidase-encoding genes *nag1* and *nag2* by using the split-marker strategy. Schematic representations of vector construction are shown in (a) for the single knockout strains, and (b) for the double knockout strains (a) Primers 1F/2R and 3F/4R amplify 1kb target gene flanking sequences. Primers 2R and 3F are hybridised once the 5′ ends complement the M13F and M13R sequences, respectively. For *nag1*, the primer pairs Chito2_50.1/Chito2_M13F and Chito2_M13R/Chito2_30.1 were used and, for *nag2,* the primer pairs HEX1_Fw/HEX1.M13F_Rev and HEX2.M13R_Fw/HEX2_Rev were used. Primer pairs M13F/HYsplit and M13R/YGsplit amplify the ‘HP’ and ‘PH’ marker fragments, respectively. Two separate PCR reactions (1F/HYsplit) and (YGsplit/4R) fuse the flanking sequences to the 5′ ‘HP’ or 3′ ‘PH’ fragments of the hygromycin resistance gene *hph*. Similar steps were used for double knockout mutant generation (b), using a *nag2* deletion mutant for transformation with a second selectable marker *ilv1* bestowing resistance to sulfonylurea. In this case, the primer pairs used in the first round PCR reactions were Chito2_M13R/Chito2_30.1 and Chito2_50.1/Chito2_M13F for amplification of the 1 kb *nag1* flanking sequences and ILsplit/M13R and M13F/LVsplit for amplification of the ‘IL’ and ‘LV1’ fragments, respectively. Second round PCR reactions were performed with the primer pairs Chito2_30.1/ILsplit and LVsplit/Chito2_50.1. Primer sequences are shown in Table 1. (c), Putative ?*nag2* transformants. The lane indicated by Wt consists of wild type *Tv.*223 DNA and lanes 1–14 contain DNA of putative ?*nag2* knockouts. The ?*Tvnag2* mutant in lane 10 (confirmed by the absence of a band) was selected for *nag2* loss-of-function studies and for the generation of ?*Tvnag1*?*Tvnag2* mutants. (d) Putative ?*nag1* transformants. The lane indicated by Wt consists of wild type *Tv.*223 DNA and lanes 1–13 contain DNA of putative ?*nag1* knockouts. The ?*Tvnag1* mutant in lane 5 (confirmed by the absence of a band) was selected for *nag1* loss-of-function studies. (e) Putative ?*Tvnag1*?*Tvnag2* transformants. Lane 1 contains DNA of the ?*Tvnag2* mutant (lane 10 in (c)) used for the generation of the double mutants. Lane 2 contains DNA of the ?*Tvnag1* mutant from lane 5 in (d). Lanes 3–12 contain DNA of putative double mutants. The double mutant, ?*Tvnag1*?*Tvnag2*, in lane 3 was selected for loss-of-function studies.

Two different strategies were used for generation of chitinase-deficient mutants. Targeted gene deletion by the split-marker method [53] was used for deletion of the two *N*-acetyl-ß-D-glucosaminidase-encoding genes, *Tvnag1* and *Tvnag2*. In addition, a double mutant (Δ*Tvnag1*Δ*Tvnag2*) was generated in which both *N*-acetyl-ß-D-glucosaminidase-encoding genes were deleted in a single strain. The constructs were obtained using fusion PCR from two rounds of PCR reactions (Figure 1). Selectable marker primers and gene-specific primers used to make the constructs are listed in Table 1.

**Figure 2:**
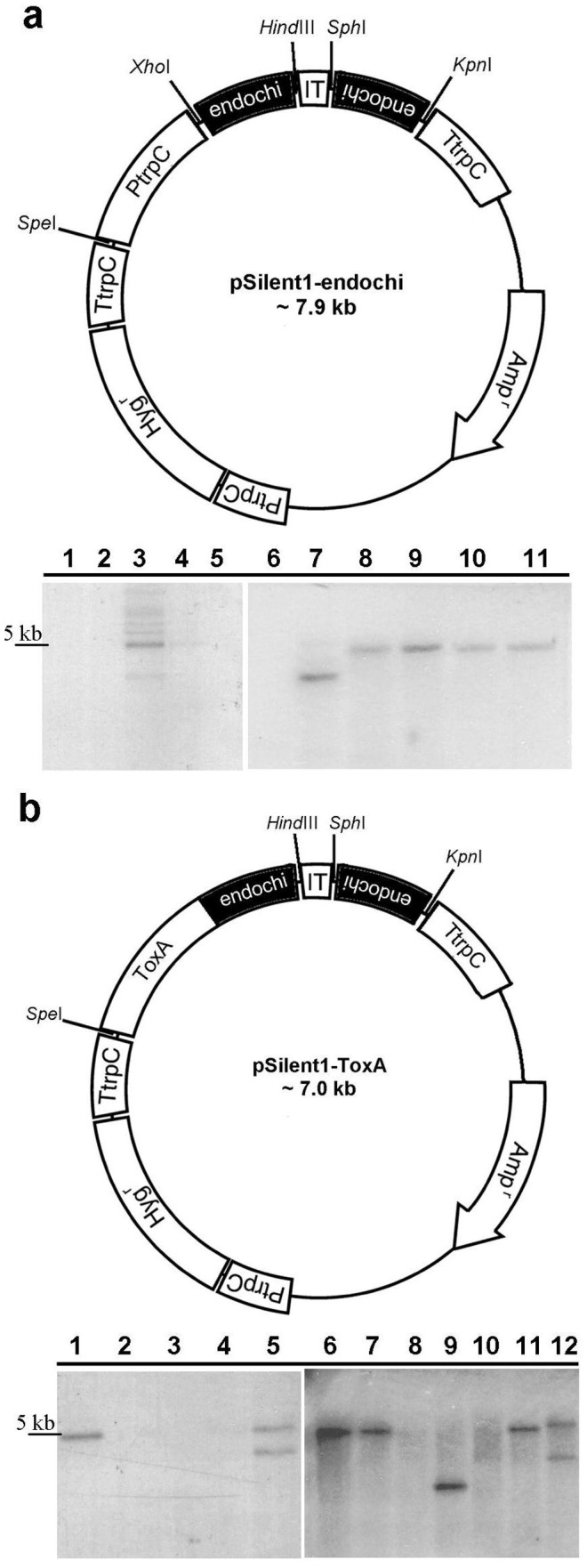
RNA-mediated silencing vectors and Southern blot analysis. (**a**) Schematic representation of the vector pSilent1-endochi, derived from pSilent-1 (Nakayashiki *et al.*, 2005) by two asymmetric clonings of a 491 bp *Tvech2* fragment. (**b**) Schematic representation of the vector pSilent1-ToxA, derived from pSilent-1 (Nakayashiki *et al.*, 2005) by replacement of the trpC promoter by ToxA followed by two asymmetric clonings of a 491 bp *Tvech2* fragment. Amp^r^, ampicillin-resistant gene; Hyg^r^, hygromycin-resistant gene; endochi, 491 bp *Tvech2* fragment; IT, intron 2 of the cutinase (CUT) gene from *Magnaporthe oryzae*; PtrpC, *Aspergillus nidulans* trpC promoter; and TtrpC, *A. nidulans* trpC terminator. Southern blots are show below the vector maps. Genomic DNA was digested with *Sac*I + *Kpn*I and probed with a 1 kb fragment amplified from the hygromycin cassette with the primers Hyg probe 2F/Hyg probe 3R. In (**a**): lane 1 contains DNA of the wild type strain *Tv.*223 and lanes 2–11 contain DNA of the putative transformants S.pNO6::*hph*, S.pNO8::*hph* (used in the loss-of-function studies), S.pNO15::*hph*, S.pNO23::*hph*, S.pNO28::*hph*, S.pNO3::*hph*, S.pNO5::*hph*, S.pNO9::*hph*, S.pNO13::*hph* and S.pNO31, respectively. In (**b**): lane 1 contains DNA of the transformant S.pOf28::*hph* (used in the loss-of-function studies), lane 2 contains the DNA of the wild type strain *Tv.*223 and lanes 3–12 contain DNA of the putative transformants S.pOf31::*hph*, S.pOf32::*hph*, S.pOf39::*hph*, S.pOf11::*hph*, S.pOf21::*hph*, S.pOf17::*hph*, S.pOf36::*hph*, S.pOf40::*hph*, S.pOf15::*hph* and S.pOf8::*hph*, respectively. The presence of a band confirms insertion of the vector.

Given the redundancy of endochitinase-encoding genes in the *T. virens* genome, we used conditional gene silencing by RNA interference (RNAi) [54] as a strategy for obtaining simultaneous silencing of all endochitinase-encoding genes. Selection of target chitinase genes to be silenced was based on a similarity analysis of the eight *T. virens* subgroup A chitinases (data not shown), in which we selected the three most similar gene sequences, the chitinases *Tvech1, Tvech2* and *Tvech3*. Two silencing vectors (pSilent-endochi and pSilent-ToxA) (Figure 2) were constructed using the silencing vector pSilent-1 as a template. The pSilent-1 vector carries a hygromycin resistance cassette and a transcriptional unit allowing double-stranded RNA expression caused by splicing of a cutinase gene intron from *Magnaporthe oryzae* [54]. For construction of gene silencing vectors, a 491-bp fragment from *Tvech2* was targeted for silencing. This fragment represents the most conserved region shared by the three chitinase gene sequences and was amplified from *Tv.*223 genomic DNA with two pairs of primers: 1F.endochi.XhoI +2R.endochi.HindIII and 3F.endochi.KpnI +4R.endochi.SphI (Table 1). The PCR fragments (1F2R and 3F4R) were eluted from the gel (Wizard® SV Gel and PCR Clean-up System, Promega) and cloned into the plasmid pGEM-T (Promega). Plasmid DNA from bacterial colonies containing pGEM-T-1F2R and pGEM-T-3F4R were digested with the restriction enzymes *Xho*I + *Hind*III and *Kpn*I + *Sph*I, respectively. The resulting 491-bp fragments contained the restriction sites required for cloning into pSilent-1 in both sense and antisense orientations. For constructing the silencing vector pSilent-endochi (7.9 kb), the 1F2R fragment was cloned into the *Xho*I site of the vector pSilent-1 (6.9 kb) downstream of the *trp*C promoter. Subsequently, the fragment 3F4R was inserted into the *Sph*I site downstream of the cutinase gene intron. The pSilent-ToxA (7.0 kb) vector was constructed by cloning the 3F4R fragment into the *Sph*I site of the vector pSilent-1, and then replacing the *trp*C promoter (1.6 kb) by the fusion fragment (ToxA+1F2R) at the *Spe*I restriction site. The fusion fragment was generated by two rounds of PCR reactions; in the first round the bacterial promoter ToxA and the 1F2R fragment were amplified using the primer pairs 1F.SpeI.ToxA +2R.fusion.ToxA and 3F.fusion.ToxA +2R.endochi.HindIII, respectively; in the second round PCR the first round products and the 1F.SpeI.ToxA +2R.endochi.HindIII primer pair were used to amplify a 1 kb fragment containing ToxA promoter linked to the 1F2R fragment. This fragment was eluted from the gel (Wizard® SV Gel and PCR Clean-up System, Promega) and cloned in the plasmid pGEM-T (Promega).

### Fungal Transformations

Preparation of protoplasts and polyethylene glycol-mediated transformation of *T. virens* 223 was performed as described previously [55]. For double gene deletion selection, the protoplasts were plated onto 0.8 M sucrose BDCM (yeast nitrogen base without amino acids (Difco), 1.7 g l^−1^, asparagine 2 g l^−1^, NH_4_NO_3_ 1 g l^−1^, glucose, 10 g l^−1^, pH to 6.0 with Na_2_HPO_4_), grown for 24 h at 26°C in the dark, and then covered with a 15 ml BDCM overlay containing 300 µg ml^−1^ of sulfonylurea (Chlorimuron ethyl, Chem Service). For all other transformations, the protoplasts were plated onto PDA containing 0.8 M sucrose, grown for 24 h at 26°C in the dark and covered with a 15 ml PDA overlay containing 900 µg ml^−1^ of hygromycin B (Calbiochem). Putative transformants were transferred to fresh selective medium and allowed to sporulate. Single spores were isolated from each putative transformant and grown in potato dextrose broth (PDB, Difco) to allow production of mycelia for DNA extraction [50]. The gene deletion events were identified by Southern blot analysis using, as a probe, a 1-kb fragment amplified with primers that anneal within the gene cassette. The insertion of the gene silencing vectors (pSilent1-endochi and pSilent1-ToxA) into the *Tv.*223 genome was confirmed by Southern blot using, as a probe, a 1-kb fragment amplified from the hygromycin cassette. The primers used are listed in Table 1. Gel electrophoresis, restriction enzyme digestions and DNA gel blot hybridizations were performed according to standard procedures [56]. Probes were radiolabelled with P^32^ using the random primer method [57].

### Gene Silencing

The *T. virens* wild type strain *Tv.*223 and twelve putative silenced mutants (transformed with the pSilent-endochi or the pSilent-ToxA silencing vectors) were subjected to five rounds of subculturing on PDA, following by subculturing on PDA containing hygromycin. Stable transformants were grown in 100 ml of PDB, inoculated with 1×10^8^ conidia m l^−1^, for 6 d at 26°C with shaking (120 rpm) and transferred to 100 ml of chitin minimal medium (CMM; crab shell chitin (Sigma), 10 g l^−1^ NaNO_3_, 6 g l^−1^, KCl, 0.5 g l^−1^, KH_2_PO_4_ 1.5 g l^−1^, MgSO_4_.7 H_2_O 0.5 g l^−1^, ZnSO_4_ 0.001 g l^−1^, FeSO_4_ 0.001 g l^−1^, thiamine 0.001%, biotin 0.000025%, pH to 6.5 with NaOH). After 24 h, the mycelium was collected and RNA isolated using the LiCl-RNA protocol. The RNA blots were performed using standard procedures [56] and probed with three different fragments, corresponding to 1-kb bands amplified from the coding regions of each chitinase gene (see Table 1 for primers sequences).

### Chitinase Assays

Spores of the *T. virens* wild type strain *Tv*.223 and five mutant strains (?*Tvnag1*, ?*Tvnag2*, ?*Tvnag1*?*Tvnag2,* S.pNO8::*hph,* S.pOf28::*hph*) were used to inoculate PDB (1×10^6^ conidia ml^−1^) and cultures were incubated for 4 d at 26°C with shaking (120 rpm), prior to transfer to CMM. After 48 h, the mycelium was separated from the culture fluids by centrifugation at 10,000 *g* for 10 min at 4°C. Supernatant fluids were collected and stored at −20°C prior to chitinase assays. There were three replicates per treatment and negative control samples consisted of uninoculated medium only. The total protein concentration in the extracts was measured according to the Bradford method [58]. The *N-*acetyl-ß-D-glucosaminidase, chitobiosidase and chitotriosidase activities were measured by using enzymatic hydrolysis of the substrates 4-Nitrophenyl N-acetyl-ß-D-glucosaminide, 4-Nitrophenyl N,N’-diacetyl-ß-D-chitobioside or 4-Nitrophenyl ß-D-N,N’,N’’-triacetylchitotriose, respectively. The substrate hydrolysis releases *p-*nitrophenol (4-nitrophenol), which upon ionization in basic pH, can be measured colorimetrically at 405 nm. The enzymatic assays were performed according to the instructions provided in a commercial Chitinase Assay Kit (Sigma).

### Phenotypic Characterisation of Transformants

*T. virens Tv*.223 and the five chitinase mutants were examined for hyphal growth and spore production. Plugs of mycelium (2 mm diameter) were removed from the margins of actively growing colonies and were placed in the centre of PDA plates. Plates were incubated at 25°C under a 16 h photoperiod of fluorescent light and colony diameters were measured after 12, 24, 36 and 48 h. Spore production was determined by removing three fungal discs (5 mm diameter) from 5-day-old cultures, placing them in sterile dH_2_O, vortexing for 1 min, and counting conidia with a haemocytometer. Each treatment was replicated three times, and the entire experiment was repeated twice.

### Biological Control Assays

The biological control capabilities of *Tv.*223 and the five chitinase-deficient mutants were conducted using three different plant pathogens. These were *Ceratocystis paradoxa* (the causal agent of sugarcane pineapple disease), *Sclerotinia sclerotiorum* (a plurivorous soil-borne damping-off pathogen) and *Rhizoctonia solani* (a pre-emergence damping-off strain of the pathogen). Wheat bran inoculum was prepared by inoculating an autoclaved mixture of 10 g wheat bran and 30 ml dH_2_O with five plugs of mycelium (5 mm diameter), and allowing growth for 5 days at 25°C. Eight g of 5-day-old inoculum was mixed thoroughly with 300 g of growing medium. Sugarcane (*Saccharum* sp.) setts (cv. SP80–1842), with one bud each, were planted in Basaplant compost inoculated with both *T. virens* and the pathogen *C. paradoxa* and were evaluated weekly for percentage germination. Each treatment had 12 replicates and the entire experiment was repeated twice. Lettuce seeds (*Lactuca sativa*, cv. Webb’s Wonderful) were planted in square plates containing an autoclaved peat preparation (1 l sphagnum moss peat (Shamrock) and 400 ml dH_2_O) inoculated with bran inoculum of *T. virens* and the pathogen *S. sclerotiorum*. Each treatment consisted of 3 replicate plates each containing 25 lettuce seeds, and percentage germination was determined after 10 d. Bean seeds (*Phaseolus vulgaris* L, cv. IAC-Alvorada) were planted in Basaplant compost inoculated with *T. virens* and the pathogen *R. solani*, and percentage germination was determined after 20 d. Each treatment was replicated six times, with five seeds each replicate and the entire experiment was repeated twice. All the bioassays included controls that consisted of plants grown in uninoculated soils.

### Data Analysis

Statistical analysis of experiments was performed by the general linear models procedures of Statistical Analysis Systems (SAS Institute Inc., Cary, NC, USA). Mean separations were conducted using the *means* procedure of SAS. Percentage germination data from biocontrol assays were normalised by transformation using arc sin^−1^ function before statistical analysis.

## Results

### Generation of Chitinase-deficient Mutants of *T. virens*

We set out to functionally characterize the role of chitinolytic enzyme activity in the biocontrol fungus *T. virens* strain 223. Analysis of its genome sequence revealed the presence of five genes, including *Nag1* and *Nag2,* which encode *N-*acetyl-ß-D-glucosaminidases, and three genes that putatively encode endochitinases; *ech1, ech2,* and *ech3*. Strain 223 was transformed with specific constructs (Figure 1a) to generate deletion mutants in which the *N-*acetyl-ß-D-glucosaminidase-encoding genes *nag1* or *nag2* were replaced by a 1.4 kb cassette, comprising the *hph* gene which bestowed resistance to the antibiotic hygromycin B. Hygromycin-resistant transformants were identified by Southern blot (Figure 1c,d) and stable transformants selected. A single *nag1* deletion mutant (lane 5 in Figure 1d) was selected for further study, and is referred to hereafter as ?*Tvnag1.* A *nag2* deletion mutant (lane 10 in Figure 1c), hereafter referred to as ?*Tvnag2*, was generated and then used to produce a double mutant (hereafter referred to as ?*Tvnag1*?*Tvnag2*), in which the *nag1* gene was replaced by a 2.8 kb cassette comprising the *ilv1* gene which bestowed resistance to the selectable marker sulfonylurea (Figure 1b).

**Figure 3:**
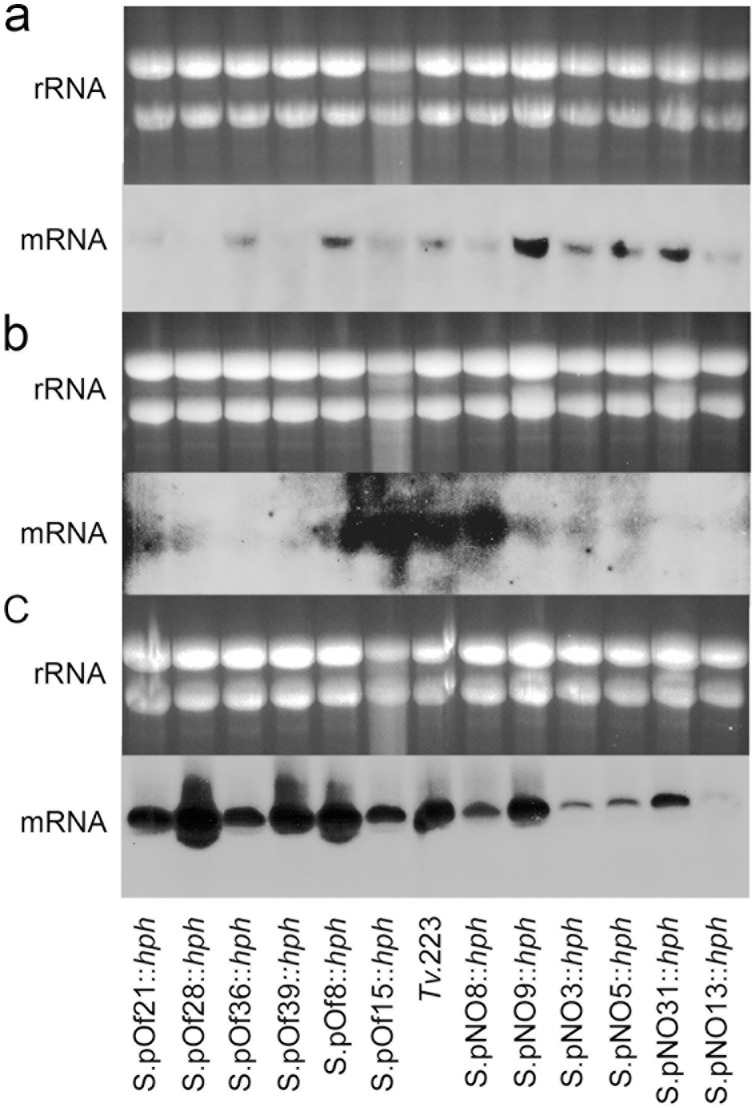
Northern blot analysis. The effects of insertion of the silencing vectors pSilent1-endochi and pSilent1-ToxA on the expression of three chitinase genes (*Tvech1, Tvech2* and *Tvech3*) in *T. virens* transformants are shown in (**a**) detection of *Tvech1* transcripts, (**b**) detection of *Tvech2* transcripts and, (**c**) detection of *Tvech3* transcripts. Transformants containing pSilent1-endochi vector have the prefix S.pNO and the ones containing pSilent1-ToxA have the prefix S.pOf. Twenty micrograms of total RNA were separated on formaldehyde-agarose gels, transferred to nylon membranes and probed with a 1 kb gene-specific fragment. Equal loading of total RNA was estimated by ethidium bromide staining of rRNA.

To carry out silencing of the endochitinase gene family, *T. virens* 223 was transformed with two different gene silencing vectors, pSilent-endochi and pSilent-ToxA, which differed only in their promoters (Figure 2a,b). Insertion of the vectors was confirmed by Southern blot analysis (Figure 2a,b) and gene silencing evaluated by assessing levels of expression of the three *T. virens* chitinase genes *Tvech1, Tvech2* and *Tvech3* by Northern blot analysis (Figure 3). The results showed different consequences from insertion of the silencing vectors in the *T. virens* genome. Total elimination, reductions, no change or even increases in chitinase mRNA transcript levels were observed in the transformants investigated (Figure 3). Only two of the transformants, S.pOf28::*hph* and S.pNO8::hph, were stable after five consecutive rounds of subculturing on PDA and hygromycin. S.pOf28::*hph* exhibited complete elimination of the *Tvech1* transcript and reduction in the *Tvech2* transcript compared to the wild type control. S.pNO8::*hph* exhibited reductions in *Tvech1* and *Tvech3* transcripts, but no reduction in *Tvech2* mRNA. Replacement of the trpC promoter (in the pSilent1-endochi vector) by ToxA promoter (in the pSilent1-ToxA vector) did not lead to a higher efficiency in the silencing of the endochitinase genes (Figure 3).

### Enzyme Activities of Chitinase-deficient Mutants

**Figure 4:**
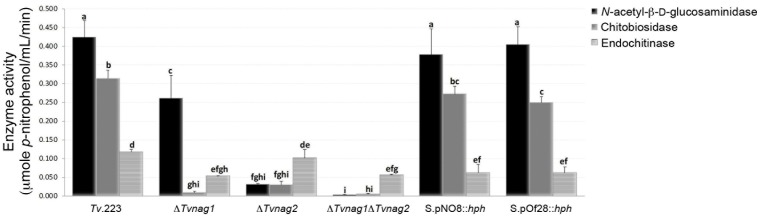
Detection of chitinolytic activities in culture filtrates of the *T. virens* strains. *N-*acetyl-ß-D-glucosaminidase, chitobiosidase and endochitinase activities of the wild type strain *Tv.*223 and the five chitinase-deficient mutants ?*Tvnag1*, ?*Tvnag2*, ?*Tvnag1*?*Tvnag2,* S.pNO8::*hph,* and S.pOf28::*hph* were determined by enzymatic hydrolysis of the chitinase-specific substrates 4-Nitrophenyl *N*-acetyl-ß-D-glucosaminide, 4-Nitrophenyl N,N’-diacetyl-ß-D-chitobioside and 4-Nitrophenyl ß-D-N,N’,N’’-triacetylchitotriose, respectively. Substrate hydrolysis releases *p*-nitrophenol which, upon ionization in basic pH, can be measured colorimetrically at 405 nm. Protein concentrations of the extracts were adjusted to 20 µg.mL^−1^ prior to enzyme assays. Histograms are the means of three replicate values with standard deviations. One unit of the activity releases 1.0 µmole of *p-*nitrophenol from the appropriate substrate per minute at pH 4.8 and 37°C. Letters denote the results of a *t*-test for comparison of means. Bars with different letter(s) are significantly different at 95% confidence level.

To test the chitinolytic activities of the five chitinase mutants (?*Tvnag1*, ?*Tvnag2*, ?*Tvnag1*?*Tvnag2,* S.pNO8::*hph,* S.pOf28::*hph*) and the wild type strain *Tv*.223 enzymatic hydrolysis of chitinase substrates was carried out. Comparison of the chitinase activities of the mutants to *Tv*.223 showed that the enzyme disruption strategies were successful in generating mutants lacking different chitinolytic activities (Figure 4). Activities of *N-*acetyl*-*ß-D-glucosaminidase were significantly reduced in the single ?*Tvnag1* and ?*Tvnag2* mutants, and completely eliminated in the double knockout ?*Tvnag1*?*Tvnag2* (Figure 4). No changes were observed in the *N-*acetyl-ß-D-glucosaminidase activities of the knock-down mutants S.pNO8::*hph* and S.pOf28::*hph* compared to the wild type strain. Chitobiosidase activities were significantly reduced in the ?*Tvnag1* and ?*Tvnag2* single mutants, and completely eliminated in the double mutant ?*Tvnag1*?*Tvnag2*. The mutants ?*Tvnag1* and ?*Tvnag1*?*Tvnag2* exhibited significant reductions in their endochitinase activities, and while a reduction in endochitinase activity was also found in the ?*Tvnag2* mutant, the decrease was not significant when compared to *Tv*.223. Endochitinase activities were significantly reduced in the two knock-down mutants, with an approximately 50% decrease in both S.pNO8::*hph* and S.pOf28::*hph* (Figure 4). *N-*acetyl*-*ß-D-glucosaminidase and chitobiosidase activities were unchanged in the mutant S.pNO8::*hph*. *N-*acetyl*-*ß-D-glucosaminidase activity was similarly unchanged in the mutant S.pOf28::*hph*, but there was a slight (albeit significant) reduction in chitobiosidase activity.

### Growth Rates and Sporulation of Chitinase-deficient Mutants

Hyphal development and sporulation of mutants was determined prior to biocontrol assays, to ensure that all strains had similar growth characteristics. The results showed no evident abnormalities in the mutants based on hyphal growth and sporulation efficiencies. All of the mutants showed similar colony morphology, growth rates, and conidiation compared to each other and to the wild type strain *Tv*.223 (Table 2).

**Table 2:** Colony diameters and conidiation of wild type *Tv.*223 and the chitinase-deficient mutants.

Strain	Colony diameter (cm)*				Conidiation (spores ml^−1^)
	12 h	24 h	36 h	48 h	
*Tv.*223	1.87	4.03	5.52	6.57	4.88×10^7^
*?Tvnag1*	1.82	4.03	5.50	6.60	5.09×10^7^
*?Tvnag2*	1.82	4.03	5.53	6.67	3.97×10^7^
*?Tvnag1?Tvnag2*	2.03	4.13	5.58	6.63	4.36×10^7^
S.pNO8::*hph*	1.75	4.00	5.53	6.73	5.09×10^7^
S.pOf28::*hph*	1.85	4.03	5.50	6.60	3.84×10^7^

* Each figure is the mean diameter of three replicate cultures.

Based on ANOVA (*P*<0.05), there were no significant differences in hyphal growth and conidiation between strains.

### Biocontrol Fitness of Chitinase-deficient Mutants

**Figure 5:**
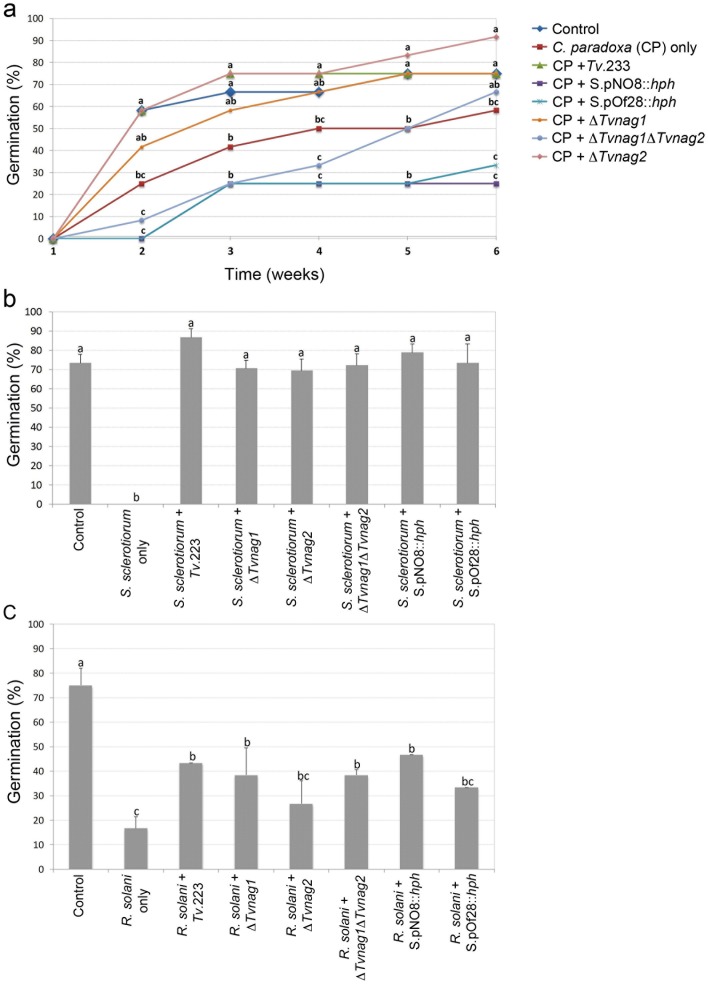
Biological control of soil-borne pathogens. (**a**) Biocontrol of pineapple disease of sugarcane caused by the soil-borne pathogen *Ceratocystis paradoxa.* Germination of sugarcane setts was determined in compost infested with the pathogen *C. paradoxa* and in mixed-species microcosms containing the pathogen and chitinase-deficient mutants. Percentage germination was measured weekly, over a 6 week period. The control consisted of sugarcane setts planted in uninfested compost. Each point is the mean of twelve replicates and percentages were converted to arc sin^−1^ values for statistical analysis by *t*-test. Points with different letters are significantly different at 95% confidence level, considering each week separately. (**b**) Biocontrol of pre-emergence damping-off disease of lettuce caused by the pathogen *Sclerotinia sclerotiorum*, Germination of lettuce seeds was determined in peat-based microcosms infested with the pathogen *S. sclerotiorum* and in mixed-species microcosms containing the pathogen and chitinase-deficient mutants. Percentage germination was measured 10 days after sowing. The control consisted of lettuce seeds planted in uninfested peat. Each point is the mean of three replicates (each consisting of 25 lettuce seed) and percentages were converted to arc sin^−1^ values for statistical analysis by Tukey test for comparison of means. Histograms with different letters are significantly different at 99% confidence level. (**c**) Biocontrol of bean rot caused by the pathogen *Rhizoctonia solani*. Germination of bean seeds was determined in compost infested with the pathogen *R. solani* and in mixed-species microcosms containing the pathogen and chitinase-deficient mutants. Percentage germination was measured 20 days after sowing. The control consisted of bean seeds planted in uninfested compost. Each point is the mean of six replicates and percentages were converted to arc sin^−1^ values for statistical analysis by Tukey test for comparison of means. Histograms with different letters are significantly different at 95% confidence level.

In order to assess whether the loss of chitinase activities altered the biocontrol capabilities of the mutants, each strain was tested for its ability to control plant diseases caused by different soil-borne pathogens. Planting sugarcane setts in soil infested with the sugarcane pathogen *C. paradoxa* led to a significant reduction (P<0.05) in the germination of plants, a trend followed throughout the 6 week sampling period. In week 2, when the buds started to germinate, there were significant reductions in germination of setts treated with the pathogen alone and with mixed populations of the pathogen and the *T. virens* mutants ?*Tvnag1*?*Tvnag2,* S.pNO8::*hph* or S.pOf28::*hph*, indicating a loss of biocontrol fitness in these mutants (Figure 5a). This effect was more persistent over the 6 week period for the mutants S.pNO8::*hph* and S.pOf28::*hph*. At week 6, germination percentages for these mutants were significantly reduced compared to wild type-treated setts. Furthermore, while not significantly different, germination percentages were also lower in the S.pNO8::*hph* + *C. paradoxa* and S.pOf28::*hph* + *C. paradoxa* treated setts than in setts treated with *C. paradoxa* only (Figure 5a). In contrast to sugarcane, lettuce infection studies showed that none of the chitinase mutants exhibited reduced capacities to control the pre-emergence damping-off pathogen *S. sclerotiorum*, when compared to the wild type strain *Tv*.223 (Figure 5b). Microcosms inoculated with *S. sclerotiorum* only exhibited total inhibition of lettuce seed germination, whereas microcosms inoculated with *Tv.*223 or the mutants and the pathogen showed various degrees of germination (from 69.3% up to 86.7%), all of which were not statistically different when compared to the control (Figure 5b). In addition to being able to protect lettuce seeds from disease, treatment with the wild type strain resulted in a higher percentage of germination when compared to the control treatment (Figure 5b). Strikingly, tests with the *T. virens* mutants and the pathogen *R. solani*, showed that none of the chitinase-deficient mutants had reduced fitness in protecting bean seeds from the pathogen when compared to the wild type strain (Figure 5c). Treatment with *Tv.*223 and the chitinase mutants resulted in statistically similar rates of germination (Figure 5c). However, it was found that while *Tv*.223 protected bean seeds from the pathogen, it was unable to restore germination rates comparable to that of the pathogen-free control experiment.

## Discussion

The relevance of chitinolytic enzymes to the biological control activities of *Trichoderma* spp. remains controversial, with conflicting evidence regarding their importance in plant disease control. The chitinolytic system of *T. atroviride* is the most comprehensively investigated system to date, although the chitinase genes of *T. virens* have also previously been described [24], [43], and one of the chitinases (*T. harzianum chit42* = *T. harzianum ech42* = *T. virens ech1*) studied in detail [9], [44]. In the present work, we investigated the involvement of *N-*acetyl-ß-D-glucosaminidases (GH family 20) and subgroup A chitinases (GH family 18) in the biocontrol fitness of a sugarcane endophytic strain of *T. virens* (strain *Tv*.223). Our objective was to determine whether these enzymes contributed to its fitness as a biocontrol agent of sugarcane pineapple disease caused by the soil-borne pathogen *Ceratocystis paradoxa*. Furthermore, we aimed to determine whether loss of chitinase activities affected its ability to control root diseases caused by other soil-borne pathogens. The presence of only two *N-*acetyl-ß-D-glucosaminidase-encoding genes in the *T. virens* genome meant that a targeted gene deletion strategy could be employed to generate single and double gene deletion mutants lacking *N-*acetyl-ß-D-glucosaminidase activities. In contrast, *T. virens* possesses 8 subgroup A chitinases and a total of 36 chitinase genes [43]. This redundancy in chitinase-encoding genes meant that an alternative strategy was needed for disruption of endochitinase activities. To this end, we used RNA interference [54] to disrupt endochitinase production by silencing the endochitinase gene *Tvech2*.

Using the split-marker method [53], we generated single (?*Tvnag1* or ?*Tvnag2*) and double (?*Tvnag1*?*Tvnag2*) deletion mutants lacking *N-*acetyl-ß-D-glucosaminidase activities. Using enzyme activity assays, we showed that the single deletion mutants had significantly reduced *N-*acetyl-ß-D-glucosaminidase activities, with total elimination in the double mutant. Elimination of both *nag1* and *nag2* genes in the ?*Tvnag1*?*Tvnag2* double mutant resulted in concomitant, and significant, loss of chitobiosidase and endochitinase activities compared to the wild-type strain. While simultaneous loss of chitobiosidase and endochitinase activities was also exhibited in the single deletion mutants, loss of chitobiosidase activities was more pronounced in both mutants compared to endochitinase activities. Loss of chitobiosidase and endochitinase activities as a consequence of *N-*acetyl-ß-D-glucosaminidase disruption is in keeping with other studies that showed that *N-*acetyl-ß-D-glucosaminidase activity in *T. atroviride* and *T. hamatum* is essential for the induction of other chitinase activities [36], [49].

Pineapple disease of sugarcane is characterized by a reduction in the germination of sugarcane setts, leading to patchy establishment of plants. We therefore chose germination assays of different host plants (sugarcane, lettuce and bean) to quantify the biocontrol capabilities of the chitinase mutants during antagonistic interactions with soil-borne, root-infecting, pathogens. The different pathobiologies of the three pathogens were reflected in the lengths of the germination assays, with bean and lettuce seeds taking substantially less time to germinate and emerge than sugarcane setts when exposed to their respective pathogens.

Despite the loss of chitinase production in the *N-*acetyl-ß-D-glucosaminidase-deficient mutants, their biocontrol activities against the sugarcane ascomycete pathogen *C. paradoxa*, and the plurivorous ascomycete and basidiomycete pathogens *Sclerotinia sclerotiorum* and *Rhizoctonia solani* respectively, were largely unaltered compared to the wild-type strain *Tv*.223. Despite an initial lag in germination of sugarcane setts in mixed populations of *C. paradoxa* and the double mutant ?*Tvnag1*?*Tvnag2*, there was no significant difference in plant establishment compared to the untreated control by the end of the sampling period. Similarly, there was no significant reduction in abilities of the mutants to control lettuce and bean diseases. The dispensable role of *N-*acetyl-ß-D-glucosaminidase to biocontrol activity was proposed previously [16] and our work supports this suggestion. Our findings are also consistent with a recent study by [59] that showed that the biocontrol efficacy of *T. atroviride* was unaffected by single and double deletions of the *nag1* and *nag2* genes.

Using RNAi, we successfully silenced the three *Tv*.223 endochitinase genes *Tvech1, Tvech2* and *Tvech3.* Different levels of mRNA accumulation were exhibited among the mutants, varying from total absence of target mRNA transcripts to no change or even an increase in mRNA accumulation in some mutants. Simultaneous silencing of *Tvech1, Tvech2* and *Tvech3* genes was detected in approximately 17% of the mutants analyzed, whereas half of the mutants exhibited silencing of two of the endochitinases. Co-silencing of genes [60]–[62] seems likely to be correlated, to some extent, with the degree of sequence similarity to the target gene.

Despite initial success with the gene silencing strategy, it was found that the majority of mutants selected were unstable. Only two of the mutants were found to be stable following sequential rounds of selection for antibiotic resistance. The two stable mutants, S.pNO8::*hph* and S.pOf28::*hph*, that differed only in the promoter employed, showed differing levels of mRNA silencing. While S.pOf28::*hph* exhibited total elimination of *Tvech1* expression and a reduction in *Tvech2* mRNA, S.pNO8::*hph* exhibited only slight reductions in *Tvech1* and *Tvech3* transcript levels. However, this is consistent with previous reports that show that knock-down mutants generated by RNA silencing, often show different levels of gene silencing [54], [62]. Despite this, and considering the presence of a further five subgroup A chitinases and a total of 36 chitinase genes in the *T. virens* genome [43], both of the mutants showed a 50% reduction in endochitinase activities.

The gene silencing strategy enabled loss of endochitinase activity on biocontrol fitness of *Tv*.223 to be determined in the absence of extensive reductions in expression of *N-*acetyl-ß-D-glucosaminidase and chitobiosidase activities. There was no effect of gene silencing on *N-*acetyl-ß-D-glucosaminidase and chitobiosidase production in S.pNO8::*hph* and only slight, albeit significant, reduction in chitobiosidase activity in S.pOf28::*hph*. Thus any reduction in biocontrol fitness of these two mutants could be largely attributed to their loss of endochitinase activities. Consequently, while phenotypic analysis of the mutants revealed no evident changes in hyphal growth or sporulation, their abilities to control pineapple disease of sugarcane were found to be significantly impaired, while biocontrol of *S. sclerotinia* and *R. solani* was unaffected.

Our findings add additional support to the importance of endochitinases in the biocontrol activities of *Trichoderma* species. Reduced biocontrol fitness as a consequence of endochitinase disruption has been described in *T. virens* and *T. atroviride* gene deletion strains [44], [63]. Furthermore, increased biocontrol activities have been shown in *T. atroviride* and *T. virens* through overexpression of endochitinases [35], [44], [64] and expression of *T. atroviride* endochitinase during mycoparasitism further demonstrates its role during antagonistic interactions [65].

The specific loss of biocontrol fitness of the *Tv*.223 endochitinase-silenced mutants in the *C. paradoxa*-sugarcane pathosystem may reflect its lifestyle as a sugarcane root endophyte. Notwithstanding this, it is clear that endochitinases contribute to its fitness as biocontrol agent of sugarcane disease, but not as an antagonist of other soil-borne plant pathogens. Furthermore, *N-*acetyl-ß-D-glucosaminidases do not appear to contribute to its fitness as a biocontrol agent of *C. paradoxa* disease or plant diseases caused by other soil-borne pathogens.
